# Impact of reducing the duration of antibiotic treatment on the long-term prognosis of community acquired pneumonia

**DOI:** 10.1186/s12890-020-01293-6

**Published:** 2020-10-07

**Authors:** Ane Uranga MD, Amaia Artaraz MD, Amaia Bilbao MD, Jose María Quintana MD, Ignacio Arriaga MD, Maider Intxausti MD, Jose Luis Lobo MD, Julia Amaranta García MD, Jesus Camino MD, Pedro Pablo España MD

**Affiliations:** 1 Department of Pneumology, Osakidetza, Universitary Hospital of Galdakao-Usansolo, Barrio Labeaga s/n, 48960 Galdakao, Bizkaia Spain; 2 Research Unit, Osakidetza, Universitary Hospital of Basurto, Bilbao, Bizkaia Spain; 3 Health Services Research on Chronic Patients Network (REDISSEC), Galdakao, Bizkaia Spain; 4 Institute of Reasearch in Health Services Kronikgune, Barakaldo, Bizkaia Spain; 5 Research Unit, Osakidetza, Universitary Hospital of Galdakao-Usansolo, Galdakao, Bizkaia Spain; 6 Department of Pneumology, Osakidetza, Universitary Hospital of Basurto, Bilbao, Bizkaia Spain; 7 Department of Pneumology, Osakidetza, Universitary Hospital of Alava, Vitoria, Alava Spain; 8 Department of Pneumology, Osakidetza, Hospital of San Eloy, Barakaldo, Bizkaia Spain

**Keywords:** Pneumonia, Duration, Antibiotic, Prognosis, Complications

## Abstract

**Background:**

The optimal duration of antibiotic treatment for community-acquired pneumonia (CAP) is not well established. The aim of this study was to assess the impact of reducing the duration of antibiotic treatment on long-term prognosis in patients hospitalized with CAP.

**Methods:**

This was a multicenter study assessing complications developed during 1 year of patients previously hospitalized with CAP who had been included in a randomized clinical trial concerning the duration of antibiotic treatment. Mortality at 90 days, at 180 days and at 1 year was analyzed, as well as new admissions and cardiovascular complications. A subanalysis was carried out in one of the hospitals by measuring C-reactive protein (CRP), procalcitonin (PCT) and proadrenomedullin (proADM) at admission, at day 5 and at day 30.

**Results:**

A total of 312 patients were included, 150 in the control group and 162 in the intervention group. Ninety day, 180 day and 1-year mortality in the per-protocol analysis were 8 (2.57%), 10 (3.22%) and 14 (4.50%), respectively. There were no significant differences between both groups in terms of 1-year mortality (*p* = 0.94), new admissions (*p* = 0.84) or cardiovascular events (*p* = 0.33). No differences were observed between biomarker level differences from day 5 to day 30 (CRP *p* = 0.29; PCT *p* = 0.44; proADM *p* = 0.52).

**Conclusions:**

Reducing antibiotic treatment in hospitalized patients with CAP based on clinical stability criteria is safe, without leading to a greater number of long-term complications.

## Background

The optimal duration of antibiotic treatment in community acquired pneumonia (CAP) is not well established: discrepancies exist between the different guidelines published to date [1–3]. In 2007, IDSA / ATS included a minimum treatment of 5 days, provided the patient remains free of fever for 48–72 h and without more than one criterion of clinical instability [4]. Recently published updated guidelines keep the same recommendation [5].

The negative impact of the overuse of antibiotics is well known. In this regard, an increase in nasopharyngeal carriers of penicillin-resistant *Streptococcus pneumoniae* has been observed with the use of low-dose beta-lactams for more than 5 days in children. The unnecessarily prolonged use of antibiotics has been associated with a greater incidence of resistance, a higher number of adverse effects, worse adherence to treatment, and a higher cost [6–9].

Several studies have been published with the aim of evaluating the safety of reducing the duration of antibiotic treatment in patients with CAP [10–13]. In a recent meta-analysis involving five clinical trials involving adults with mild-to-moderate CAP comparing the same types of antibiotic, short 3 to 7 day regimens of antibiotics were compared to 7 to 10 day regimens. The authors did not observe significant differences in terms of cure rate, mortality and adverse effects [14]. Surprisingly, a new meta-analysis comparing ≤6 day versus ≥7 day regimens, observed a lower mortality rate in the shorter compared to the long regimen group (RR 0.52, 95% confidence interval [CI] 0.33–0.82), with similar cure and relapse rates in both groups [15]. When evaluating only the most severe patients, mortality was 2.2% in the group with a shorter regimen compared to 4.7% in the long regimen group.

On the other hand, the use of certain biomarkers such as procalcitonin (PCT) has been shown to be useful in reducing the duration of antibiotic treatment [16]. Moreover, there is strong evidence that support the idea that biomarkers at admission, such as proadrenomedullin, can predict worse outcome not only al early, but also at late follow-up [17]. Recently, our working group published the positive results [18] of a clinical trial designed to validate the IDSA / ATS criteria on the duration of antibiotic treatment in patients admitted for CAP. The median number of days with antibiotic in the control group was 10 as opposed to 5 in the intervention group, while the short-term clinical cure rate was similar for both groups. However, the impact that such a reduction may have on the long-term prognosis of these patients, as well as its effect on systemic inflammation, remains unknown.

The goal of the present study is to assess the impact in the long term of a reduction of antibiotic treatment in patients admitted for CAP. The method was to evaluate complications that occurred up to 1 year later in patients that had been included in a clinical trial for the validation of the IDSA / ATS criteria for the duration of antibiotic treatment.

## Methods

### Study design

Multicentre cohort study which evaluated complications after 1 year follow-up in patients who had previously been included in a randomized clinical trial on the duration of antibiotic treatment in patients admitted for CAP. In the clinical trial intervention group, antibiotic treatment was prescribed for a minimum of 5 days and was suspended if for 48 h the temperature was ≤37.8 °C and there was no more than 1 sign of clinical instability as defined by Halm’s clinical stability criteria [4]. If stability was not reached by day 5, antibiotic was stopped whenever all criteria were met, as Halm criteria were assessed every day. In the control group, the doctor decided on the duration of the antibiotic treatment. In both, control and intervention groups, antibiotic type was chosen empirically by physicians according to local clinical guidelines. The follow-up period of the original clinical trial patients was 30 days, while the present study extended the follow-up period from 30 days to 1 year.

All patients were informed about the study and asked to give their informed consent. The project was approved by the Basque Country Ethics Committee (2011–001067-51).

### Study patients

All adult patients (≥18 years) admitted for CAP and included in the clinical trial were included. Pneumonia was defined as a pulmonary infiltrate on chest X-ray not known to be old, and with symptoms indicative of pneumonia, such as cough, dyspnea, fever, and / or pleural pain. All patients previously excluded from the clinical trial due to infection by the human immunodeficiency virus were excluded, as well as the immunosuppressed (those with organ transplants or with splenectomy, those treated with 10 mg/day of prednisone or equivalent for more than 30 days or with other immunosuppressive agents, neutropenic patients), those hospitalized in the previous 14 days, patients who received prior antibiotic treatment in the previous 30 days and institutionalized patients.

The study also excluded cases of pneumonia caused by infrequent agents (eg *P.aeruginosa, S. aureus*), infectious processes that required prolonged treatment with antibiotics (i.e. bacterial endocarditis, abscesses), pneumonia with pleural effusion that required drainage, those who died or who were admitted to the Intensive Care Unit before randomization and those who did not give their informed consent.

### Data collection

At baseline, both demographic and clinical variables were collected for each patient. Severity was assessed using the PSI scale (pneumonia severity index) [19]. Comorbidity was collected using the Charlson comorbidity index [20] Vital signs were collected daily to assess clinical stability. Originally follow-up for all patients took place for up to 30 days, this period was extended to 1 year in this new study.

The main outcome variables that this study assessed were mortality at 90 days, 180 days, and 1 year, as well as new admissions for any reason that took place after the 30-day clinical trial follow-up and up to 1 year of index admission. Similarly, the occurrence of cardiovascular events was assessed during that same period of time, defined as the occurrence of hypertension, cardiac arrhythmia, valvulopathy, heart failure, coronary heart disease, decompensation of previous heart disease, intermittent claudication, thrombosis, embolism or stroke. The principal investigator at each hospital reviewed the medical records to confirm the occurrence of complications, in addition to conducting phone consultations when considered necessary. Patients or the public were not involved in the design, or conduct, or reporting, or dissemination plans of our research.

On the other hand, a subanalysis was carried out in one of the hospitals, where biomarker levels were measured at admission, at 5 days and at 30 days. It should be highlightened that the intervention was carried out at day 5, hence, the possible impact that a shortened antibiotic treatment could have on inflammation, should appear at day 30. C reactive protein (CRP) levels quantified by immunoturbidimetry with an analytical sensitivity of 1 mg/L were analyzed. Procalcitonin (PCT) was analyzed via electrochemiluminescence, with an analytical sensitivity of 0.02 ng/mL and 5 pg/mL respectively. On the other hand, proadrenomedullin (ProADM) was analyzed via sandwich immunoassay using TRACE (time-resolved amplified cryptate emission) technology with an analytical sensitivity of 0.05 nmol/L.

### Statistical analysis

For the descriptive analysis, frequencies and percentages were used for the qualitative variables, and mean and standard deviation (SD) or median and interquartile range (IQR) for the quantitative variables. Baseline characteristics of the intention to treat (ITT) population were compared between the control group and the intervention group. In addition, the main clinical outcomes up to 1 month and up to 1 year follow-up in the per protocol population (PP) were compared between the two groups. The main events were also compared between the different participating hospitals. To compare the qualitative variables the Chi-square test or Fisher’s exact test were used, whereas the t-test or Wilcoxon’s non-parametric test were used to compare the quantitative variables. Finally, the main outcomes were compared between the control group and the intervention group, adjusting for the Charlson comorbidity index, using the logistic regression model. Kaplan-Meier curves were drawn for one-year mortality in each group of patients and these were compared using the log-rank test.

Biomarker levels were compared between the two groups of patients on days 1, 5 and 30, as well as the difference from day 5 to day 30 when both were available, using the Wilcoxon non-parametric test. Biomarker differences from day 5 to day 30 were also compared between the two groups of patients, adjusting for biomarker levels on day 5, using the general linear model. On the other hand, the effect of the difference in biomarkers from day 5 to day 30 on cardiovascular events at 1 year follow-up in the per PP was analyzed, adjusting for biomarker levels on day 5 and the Charlson index, using the logistic regression model. Finally, we analyzed if this effect was different depending on the group. For this analysis, the logistic regression model was also used, considering the cardiovascular event as the dependent variable, and as independent variables: the difference in biomarker from day 5 to day 30, the group (intervention vs. control), the interaction between the difference and the group, as well as the adjustment variables for the Charlson index and the biomarker on day 5.

All results were considered statistically significant for *p* < 0.05. Analyses were performed using SAS for Windows, version 9.2 (SAS Institute, Cary, NC) and S-Plus 2000 (MathSoft Inc., Seattle, WA, 1999).

## Results

**Fig. 1:**
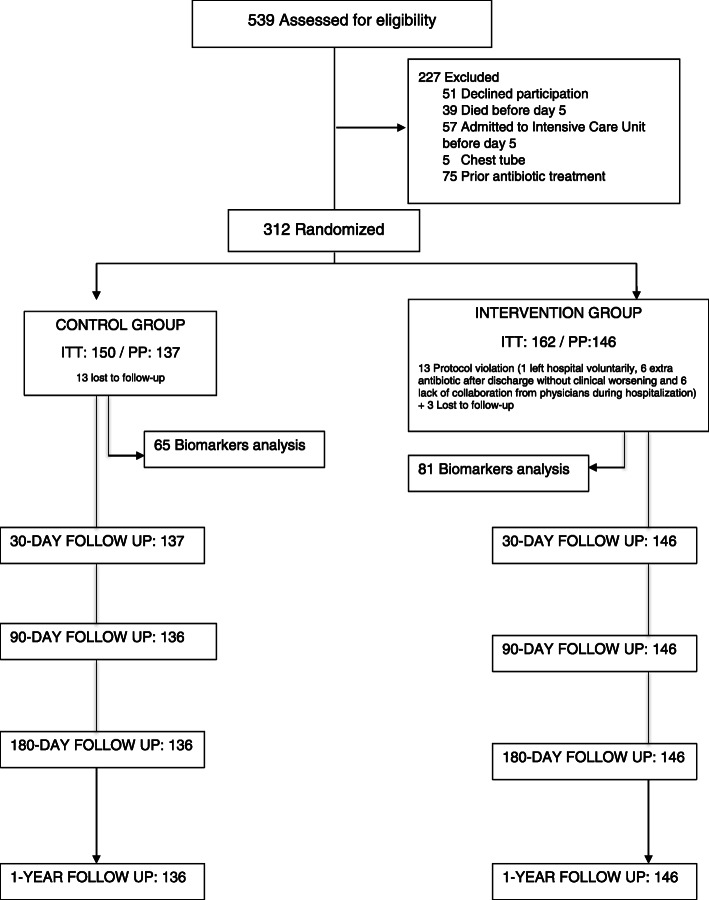
Study flow diagram. Patients available at each follow-up. ITT: Intention-to-treat, PP: Per-Protocol

A total of 312 patients were included, 150 from the control group and 162 from the intervention group (Fig. 1). Thirteen patients presented protocol violation in the intervention group. Likewise, 13 patients were lost during follow-up in the control group and 3 in the intervention group, leaving a total of 137 patients in the control group and 146 in the intervention group, in the per protocol analysis. Table 1 shows the baseline characteristics of the participants. 109 (79.56%) and 117 (80.14%) patients received quinolones alone or in combination, in the control and the intervention group, respectively. 10 (7.30%) and 13 (8.90%) patients received beta-lactams plus macrolides, in the control and the intervention group, respectively, whereas 18 (13.14%) and 16 (10.96%) patients received Beta-lactams alone, in the control and the intervention group, respectively (*p* value = 0.78).

**Table 1:** Baseline characteristics of the patients

	Control group (***n*** = 150)	Intervention group (***n*** = 162)	***p*** value
**Age**, average (SD)	66.27 (17.9)	64.72 (18.7)	0.46
**Sex,** n (%)			0.86
Male	95 (63.3)	101 (62.3)	
Female	55 (36.7)	61 (37.7)	
**Comorbidities,** n (%)			
Liver disease	4 (2.7)	4 (2.5)	1.00
Heart disease	38 (25.3)	39 (24.1)	0.80
Congestive heart failure	14 (9.3)	12 (7.4)	0.54
Cerebrovascular disease	16 (10.7)	9 (5.6)	0.10
Kidney disease	12 (8.0)	12 (7.4)	0.84
COPD	21 (14)	27 (16.7)	0.51
Diabetes mellitus	25 (16.7)	21 (12.0)	0.36
**Charlson Index,** median (IQR)	1 (0–2)	1 (0–2)	0.35
**Charlson Index, categorized,** n (%)			0.40
0	61 (40.7)	70 (43.2)	
1	37 (24.7)	47 (29.0)	
> 1	52 (34.7)	45 (27.8)	
**Categorized PSI,** n (%)			0.51
I-III	89 (59.3)	102 (63)	
IV-V	61 (40.7)	60 (37.0)	
**PSI**, average (SD)	83.7 (33.7)	81.8 (33.8)	0.63

Data are presented as average (SD), median (IQR) or n (%)

*SD* Standard Deviation, *IQR* Interquartile range

**Fig. 2:**
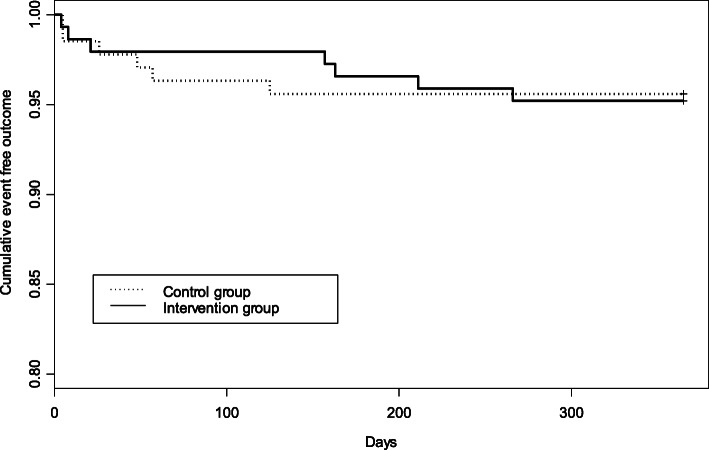
Kaplan-Meier Curves for 1-Year Mortality in Per-Protocol Analysis. The log-rank test did not show significant differences between both groups (control group with conventional treatment and intervention group with duration of antibiotic treatment based on IDSA / ATS); Hazard ratio (95% confidence interval) =1.08 (0.36, 3.22), *p* = 0.89

Table 2 shows the main results in the control and intervention groups. The overall mortality at 90 days, 180 days and 1 year per protocol was 8 (2.57%), 10 (3.22%) and 14 (4.50%), respectively. There were no significant differences in mortality after 1 year between both groups, both unadjusted and when adjusted for the Charlson comorbidity index (PP: OR adjusted 1.04, 95% CI 0.33–3.26, *p* = 0.94). The rate of new admissions per year in the control group was 27.01% while it was 25.52% in the intervention group, *p* = 0.84. The same analysis was performed for each of the hospitals without showing any significant differences. Figure 2 shows the analysis of 1-year survival by per protocol (Hazard ratio (95% CI) =1.08 (0.36, 3.22), *p* = 0.89) with no significant differences detected. On the other hand, 16 (10.74%) cardiovascular events per year were observed in the control group and 23 (14.38%) in the intervention group (adjusted OR 1.40, 95% CI 0.71–2.77, *p* value = 0.33) in the per protocol analysis (Table 2).

**Table 2:** Main results in the control group (conventional treatment) and in the intervention group (duration of antibiotic treatment based on IDSA / ATS), in the per-protocol analysis

	Control	Intervention	OR (IC 95%)^a^	***p*** value
90 day Mortality	5 (3.68)	3 (2.05)	0.48 (0.11–2.19)	0.35
180 day Mortality	5 (3.68)	5 (3.42)	0.85 (0.23–3.12)	0.80
1 year Mortality	6 (4.41)	7 (4.79)	1.04 (0.33–3.26)	0.94
1 year Admissions	37 (27.01)	37 (25.52)	0.95 (0.55–1.64)	0.84
1 year CV events	14 (10.29)	21 (14.58)	1.50 (0.73–3.08)	0.27

Data are presented as n (%)

*OR* odds ratio, *IC* confidence interval, *CV* cardiovascular

^a^OR is estimated by considering the control group as the reference group and adjusting for the Charlson comorbidity index

A sample was obtained for biomarker analysis from 65 patients in the control group and 81 in the intervention group, all from one of the hospitals. The biomarker levels at admission, day 5, and day 30 as well as the difference from day 5 to day 30 are shown in Table 3. In the per protocol analysis, after adjusting for the value at day 5, no significant differences were observed between the control and intervention groups with respect to the difference in proadrenomedullin levels from day 5 to day 30 (*p* = 0.52). Neither were significant differences detected for the CRP (*p* = 0.2910) or in the PCT (*p* = 0.44).

**Table 3:** Biomarker levels in the control group (conventional treatment) and in the intervention group (duration of antibiotic treatment based on IDSA / ATS), in the per-protocol analysis

	Control ***n*** = 61	Intervention ***n*** = 75	***p*** value
ProADM day 1	1.01 (0.78, 1.32)	0.91 (0.68, 1.25)	0.41
ProADM day 5	0.81 (0.57, 1.01)	0.81 (0.54, 1.19)	0.59
ProADM day 30	0.70 (0.49, 1.01)	0.68 (0.49, 0.93)	0.93
ProADM difference from day 5 to day 30	−0.07 (−0.23, 0.01)	−0.09 (−0.15, 0.03)	0.65
PCR day 1	240.20 (86.80, 301.30)	159.75 (87.75, 302.60)	0.47
PCR day 5	47.50 (25.10, 88.50)	37.10 (15.70, 79.30)	0.20
PCR day 30	2.55 (1.60, 7.80)	2.50 (1.30, 5.40)	0.48
PCR difference from day 5 to day 30	−42.30 (−86.70, −21.90)	−32.45 (−73.35, −14.50)	0.17
PCT day 1	0.67 (0.18, 3.34)	0.49 (0.15, 1.68)	0.67
PCT day 5	0.19 (0.09, 0.50)	0.17 (0.06, 0.75)	0.87
PCT day 30	0.04 (0.02, 0.06)	0.04 (0.03, 0.06)	0.54
PCT difference from day 5 to day 30	−0.12 (−0.40, −0.04)	−0.18 (− 0.69, − 0.03)	0.49

Data are presented as median (IQR)

*IQR* interquartile range

Last, the effect of change over time in biomarker values on the rate of cardiovascular events at follow-up was assessed (Table 4). Twenty-two cardiovascular events were observed in this patient sample. Changes in proADM values from day 5 to day 30 showed an OR of 1.11 (95% CI: 0.09, 13.65) () of having any cardiovascular event, adjusted for its value on day 5 and by the Charlson index (*p* = 0.94). The same analysis was performed taking into account the control and intervention groups, and again no significant effect was detected for cardiovascular events at 1 year in either the control or intervention group (proADM difference: *p* = 0.79 and *p* = 0.86, respectively; PCR difference: *p* = 0.38 and *p* = 0.20, respectively; and PCT difference: *p* = 0.94 and *p* = 0.63, respectively).

**Table 4:** Effect of biomarker level differences from day 5 to day 30 on cardiovascular events, by per protocol population

	CV Events	
	OR (95% CI)^**a**^	***p*** value
**ProADM Difference**	1.43 (0.11, 18.56)	0.79
**PCR Difference**	1 (0.97, 1.02)	0.80
**PCT Difference**	0.09 (0.0003, 30.82)	0.42

*OR* odds ratio, *IC* confidence interval, *CV* cardiovascular

^a^OR is adjusted for the Charlson comorbidity index and the biomarker level on the 5th day

## Discussion

The main value of the current study is that it shows the medium and long-term safety of reducing the duration of antibiotic treatment in patients admitted for a case of CAP, based on clinical stability criteria, without leading to a greater number of long-term complications; nor did it lead to higher mortality or readmission rates, nor differences in the systemic inflammation presented by these patients. That is, the fact that there are no significant long-term differences in the main results under study between the control and intervention groups, validates our proposal to reduce the duration of antibiotic treatment in patients with clinically stable CAP from the point of view of the long-term safety of the patient.

The beneficial effects of reducing the duration of antibiotic treatment have been studied widely. On the one hand, it reduces antimicrobial resistance, possible adverse effects and costs, while, on the other hand, it improves adherence to treatment [6–9]. However, despite current evidence avoiding unnecessarily prolonged treatments remains an arduous task, likely due to a false sense of security provided by longer-term treatments [21]. In fact, a retrospective study carried out in the United States in patients admitted for CAP, observed that the average duration of antibiotic treatment exceeded the recommended time by 74 and 71% for patients aged 18–64 years and ≥ 65 years, respectively [22].

A remarkable strength of this study is that it is based on a clinical trial with a unique design where the doctor him or herself decided on the type of antibiotic and in which similar cure rates were obtained for both groups. Likewise, unlike most of the studies published so far and despite the exclusion of patients requiring admission to Intensive Care, up to 40% of patients with IV and V PSI were included. However, the evidence for critically ill patients is limited. Chastre et al. carried out a double-blind clinical trial in patients with ventilator-associated pneumonia in which they compared 8-day versus 15 day antibiotic regimens [23]. The authors observed no differences between the two groups except in the case of non-fermenting gram-negative germs. Recently, in a meta-analysis in which they compared regimens of ≤6 days versus ≥7 days with similar results, they carried out a sub-analysis in patients with severe pneumonia, observing lower mortality in the group with the shorter regimen (2.2% vs. 4.7%) [15].

CAP has a great impact on systemic inflammation, both in the short and the long term [24]. PCT has been the most widely studied biomarker for reducing antibiotic treatment. De Jong et al.conducted a clinical trial in critically ill patients in which the antibiotic was discontinued if the PCT value decreased by at least 80% or below 0.5 μg / L25 [25]. The median number of days on antibiotics was 5 in the PCT group versus 7 days in the control group. Furthermore, the 1-year mortality in the PCT group was 36% as opposed to 43% in the control group (absolute difference 7.4, 1.3–13.8, *p* = 0.0188). However, the biomarker showing the best prognostic power for short and long-term complications in CAP has been proadrenomedullin [26–28]. To this effect, in our study we were able to obtain a sample for biomarker analysis from 146 patients, without observing differences in biomarker levels between the control and intervention groups.

Mortality at 1 year after a case of CAP is high and it is thought that the cause may lie in a state of persistent chronic inflammation that leads to a greater number of cardiovascular events and higher long-term mortality [29–33]. Undoubtedly, knowing the kinetics of biomarkers is crucial to measure the evolution of inflammation, proADM being the one biomarker that has shown the best results [26, 34]. For that reason, the evolution of biomarkers from day 5 to day 30 was analyzed to assess the impact of the intervention on inflammation and consequently, on cardiovascular events at late follow-up. There were not significant differences between both groups and importantly, data was adjusted by baseline value, which supports the idea that the reduction of antibiotic treatment does not impact systemic inflammation neither at late follow-up.

Finally, our study has some limitations. First, data collection from 30 days to 1 year was done retrospectively. Second, few complications were observed in the sample with biomarkers, probably due to the small sample size. Third, most patients received quinolones. Hence, we do not know if this may have influenced results. Fourth, we were not able to assess causes of death. Fifth, the results cannot be extrapolated to the excluded population. Future studies assessing patients with those characteristics are necessary.

## Conclusions

In conclusion, our study indicates that individualizing and reducing the duration of antibiotic treatment in patients with CAP based on clinical stability criteria is safe, without leading to a greater number of long-term complications or differences in systemic inflammation.

## Data Availability

The datasets used and/or analysed during the current study are available from the corresponding author on reasonable request.

## Abbreviations

CAP: Community-acquired pneumonia
PSI: Pneumonia severity index
CRP: C reactive protein
PCT: Procalcitonin
ProADM: Proadrenomedullin
PP: Per protocol
