# Correction to: Impact of reducing the duration of antibiotic treatment on the long-term prognosis of community acquired pneumonia

**DOI:** 10.1186/s12890-020-01378-2

**Published:** 2021-01-11

**Authors:** Ane Uranga, Amaia Artaraz, Amaia Bilbao, Jose María Quintana, Ignacio Arriaga, Maider Intxausti, Jose Luis Lobo, Julia Amaranta García, Jesus Camino, Pedro Pablo España

**Affiliations:** 1 Department of Pneumology, Osakidetza, Universitary Hospital of Galdakao-Usansolo, Barrio Labeaga s/n, 48960 Galdakao, Bizkaia Spain; 2 Research Unit, Osakidetza, Universitary Hospital of Basurto, Bilbao, Bizkaia Spain; 3 Health Services Research on Chronic Patients Network (REDISSEC), Galdakao, Bizkaia Spain; 4 Institute of Research in Health Services Kronikgune, Barakaldo, Bizkaia Spain; 5 Research Unit, Osakidetza, Universitary Hospital of Galdakao-Usansolo, Galdakao, Bizkaia Spain; 6 Department of Pneumology, Osakidetza, Universitary Hospital of Basurto, Bilbao, Bizkaia Spain; 7 Department of Pneumology, Osakidetza, Universitary Hospital of Alava, Vitoria, Alava Spain; 8 Department of Pneumology, Osakidetza, Hospital of San Eloy, Barakaldo, Bizkaia Spain

## Correction to: BMC Pulm Med 20, 261 (2020) 10.1186/s12890-020-01293-6

Following publication of the original article [1], the authors flagged that their article had published with ‘MD’ erroneously included in the names of all the authors.

This has since been corrected in the published article and the corrected names may be found in this correction.

The publisher apologizes for any inconvenience caused.
